# Anti-inflammatory activity of berberine in non-alcoholic fatty liver disease via the Angptl2 pathway

**DOI:** 10.1186/s12865-020-00358-9

**Published:** 2020-05-19

**Authors:** Zengsheng Lu, Beihui He, Zhiyun Chen, Maoxiang Yan, Liyan Wu

**Affiliations:** 1 Department of Infectious Diseases, Tongde Hospital of Zhejiang Province, 234 Gucui Road, Zhejiang, 310012 Hangzhou China; 2 Key Laboratory of Diagnosis and Treatment of Circulatory Diseases in Combination with Traditional Chinese and Western Medicine of Zhejiang Province, Zhejiang Hospital of Traditional Chinese Medicine, 54 Youdian Road, Zhejiang, 310006 Hangzhou China; 3 Department of Gastroenterology, Tongde Hospital of Zhejiang Province, 234 Gucui Road, Zhejiang, 310012 Hangzhou China

**Keywords:** Nonalcoholic fatty liver disease, Berberine, Angptl2, Inflammatory response

## Abstract

**Background:**

Nonalcoholic fatty liver disease (NAFLD) has become the most common liver disease worldwide. Recent studies have shown that the Angptl2 pathway mediated hepatic inflammatory response plays an important role in the progression of nonalcoholic fatty liver disease. Our study investigated the possible molecular mechanisms of berberine (BBR) in the treatment of the liver inflammatory response in the livers of rats with high-fat diet-induced NAFLD via the Angptl2 pathway.

**Results:**

At the end of 12 weeks, compared with the control group rats, the high-fat- diet group rats showed obvious pathological and biochemical changes. The levels of pro-infalmmatory cytokines (CCL2, TNF-α) were increased, the infiltration of inflammatory cells (CCR2) was elevated, and the hepatic mRNA and protein levels of Angptl2, NF-κB and Foxo1 were increased to different degrees. Nevertheless, following treatment with BBR, liver tissue pathology, biochemical data, and Angptl2 pathway-related genes expression were significantly ameliorated.

**Conclusions:**

Our findings demonstrate that BBR might attenuate the liver inflammatory response in the livers of rats with high-fat diet-induced NAFLD through the regulation of the Angptl2 pathway.

## Background

Nonalcoholic fatty liver disease (NAFLD), which includes nonalcoholic simple fatty liver, non-alcoholic steatohepatitis (NASH), liver fibrosis, cirrhosis, and hepatocellular carcinoma has become the most common liver disease worldwide, with a global incidence of approximately 24% [1]. The prevalence of adults with NAFLD in Shanghai and Guangdong Province, China, is approximately 15% [2, 3], and the incidence rate is increasing each year. In addition, NAFLD also promotes the progression of other systemic diseases, such as cardiovascular diseases and type 2 diabetes, among others [4, 5]. Nevertheless, the pathogenesis and clinical treatment of NAFLD have yet to be elucidated until now. Except lifestyle interventions, therapeutic approaches mainly include antioxidants (such as vitamin E) and peroxisome proliferator activated receptor agonists (such as thiazolidinediones) [6–8], but these interventions are associated with lack of organ or cell selectivity, and limited specificity, as well as side effects. Consequently, there is an urgent need to study new treatments for NAFLD. Recent studies have shown that metabolic syndrome consists of chronic, low-grade systemic inflammation, and NASH is considered to be the manifestation of metabolic syndrome in the liver [9]. Certain pro-inflammatory cytokines secreted by adipocytes and macrophages stimulate liver inflammatory responses and inflammatory cell infiltration in the liver by stimulating inflammatory signaling pathways, and participate in the development of NASH [10, 11].

Berberine (BBR) is a kind of isoquinoline alkaloid isolated from the Chinese medicinal herb *Rhizoma coptidis*, which has been used in traditional Chinese medicine (TCM) for centuries. It is well known that BBR has many pharmacological properties with respect to metabolic diseases and many other inflammatory diseases [12, 13]. Recently studies showed that BBR plays important roles in treating NAFLD, such as increasing insulin sensitivity, improving glucose and lipid metabolic disorders, regulating intestinal microbiota and alleviating oxidative stress; these findings suggest that BBR may serve as a potential drug for NAFLD [14–16]. However, studies on BBR treatment of the hepatic inflammatory response in NAFLD are still unclear. Angiopoietin-like protein 2 (Angptl2), a new secretory glycoprotein, belongs to the angiogenic-like protein family and is secreted by adipose tissue, macrophages (mainly Kuffer cells, KCs), and endothelial cells, among others [17]. Under normal conditions, Angptl2-mediated signal transduction contributes to angiogenesis and tissue damage repair [17], whereas excessive Angptl2 signaling leads to chronic inflammation, which is accompanied by obesity and metabolic syndrome [17], type 2 diabetes [18], atherosclerosis [19], and even certain tumors [20].Angptl2 activates Racl through integrins, which activates nuclear factor-kappaB (NF-κB) and inhibits κB inhibitor (IκB), and promotes the release of inflammatory mediators, such as TNF-α and CCL2, and the aggregation of inflammatory cells; these processes, in turn, lead to the development of chronic inflammation of the liver. Based on these data, our study used a high-fat diet-induced rat model of NAFLD to study whether BBR has an anti-NAFLD effect by inhibiting the hepatic inflammatory response via the Angptl2 pathway.

## Results

### BBR ameliorates hepatic steatosis and inflammation in HFD-fed rats

**Fig. 1:**
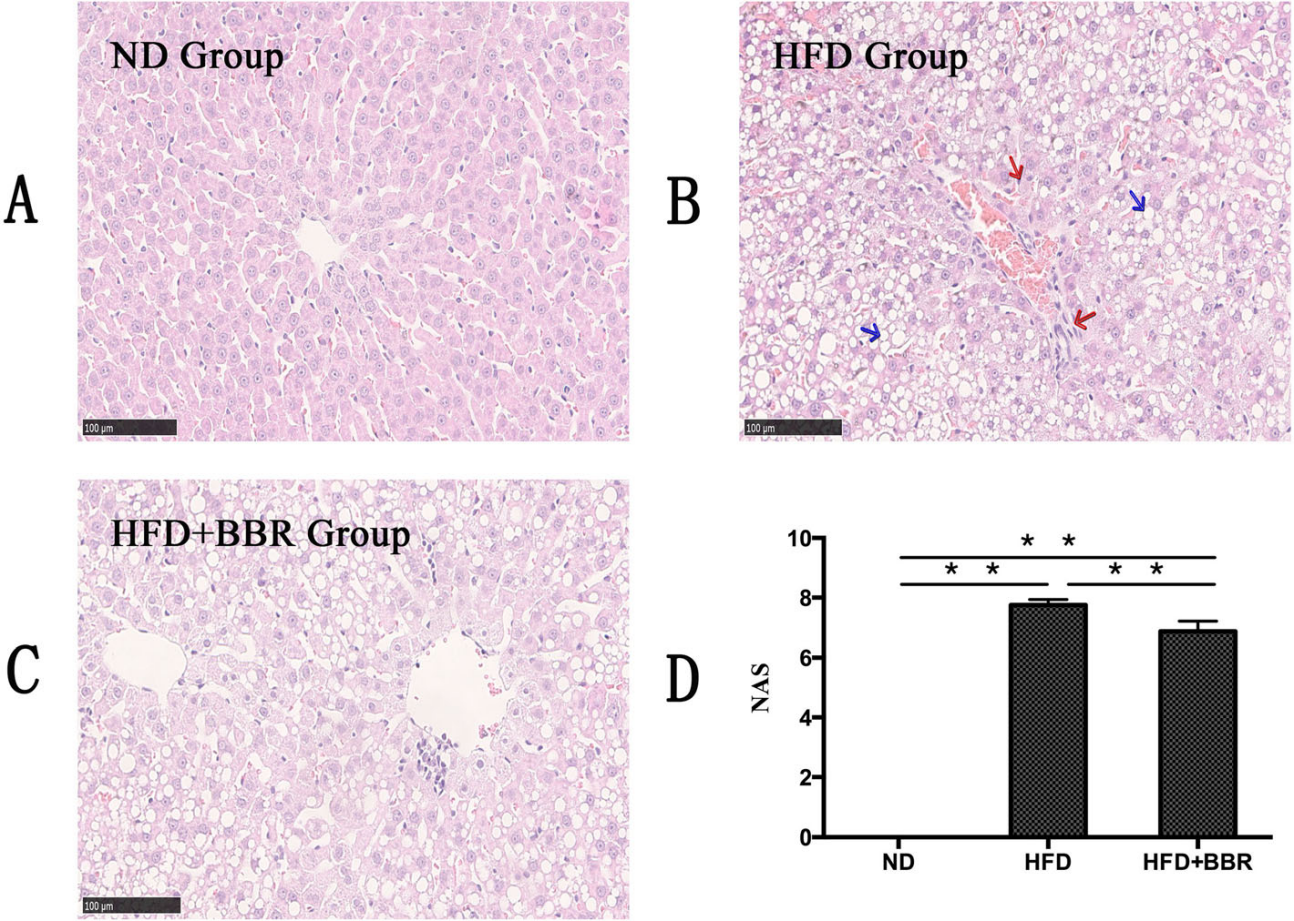
BBR ameliorates hepatic steatosis in HFD-fed rats. The liver isolated from rats fed with a normal diet (ND), high-fat diet (HFD) or high-fat plus Berberine (HFD + BBR) were stain with hematoxylin and eosin. **a** ND group; (**b**) HFD group; (**c**) HFD + BBR group. Photographs are at 100 × magnification. Steatosis shown in blue arrow, necrosis and inflamation shown in red arrows. **d** NAFLD activity score. Data are presented as mean ± SD from eight rats.**P < 0.01,* *P* < 0.05.*n* = 8/group

To confirm the therapeutic effect of BBR, we examined the effect of BBR on the liver of rats with HFD-fed induced NAFLD rats. As shown in Fig. 1, compared with those in the ND group, the liver tissues of rats in the HFD group showed obvious steatosis, inflammatory cell infiltration, and focal necrosis (Fig. 1a-c). Moreover, the NAFLD activity score (NAS) increased significantly (Fig. 1d). Compared with the HFD group, the HFD + BBR group showed a decrease in hepatic steatosis, inflammatory cell infiltration and NAS (Fig. 1).

### Biochemical changes in serum and liver in NAFLD rats

**Fig. 2:**
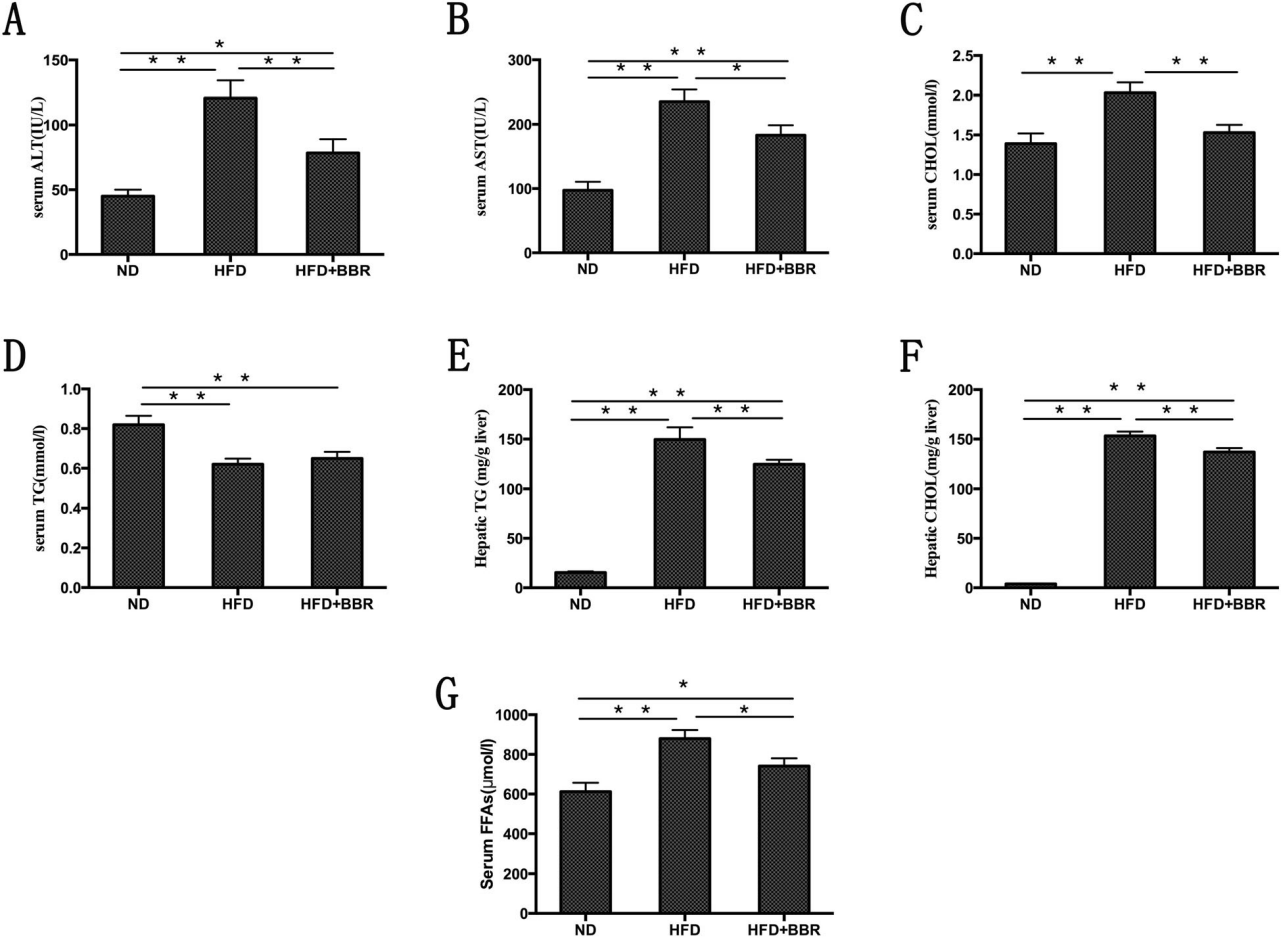
Biochemical changes in serum and liver in NAFLD rats. The level of serum ALT, AST, TG, CHOL, FFAs and hepatic TG and CHOL contents. Data are presented as mean ± SD from eight rats.**P < 0.01,* *P* < 0.05.*n* = 8/group

Serum ALT, AST, CHOL, FFAs, and liver TG and CHOL showed different degrees of increase (Fig. 2a-c and e-g). Compared with those in the HFD group, serum ALT, AST, CHOL, FFAs, hepatic TG, and hepatic CHOL in the HFD + BBR group decreased to 35.02, 22.20, 24.63, 15.74, 16.65 and 31.25%, respectively (Fig. 2a-c and e-g). Interestingly, serum TG in the HFD group showed a decrease compared with the ND group, and after the intervention with BBR, serum TG was increased (Fig. 2d).

### Serum cytokines in NAFLD rats

**Fig. 3:**
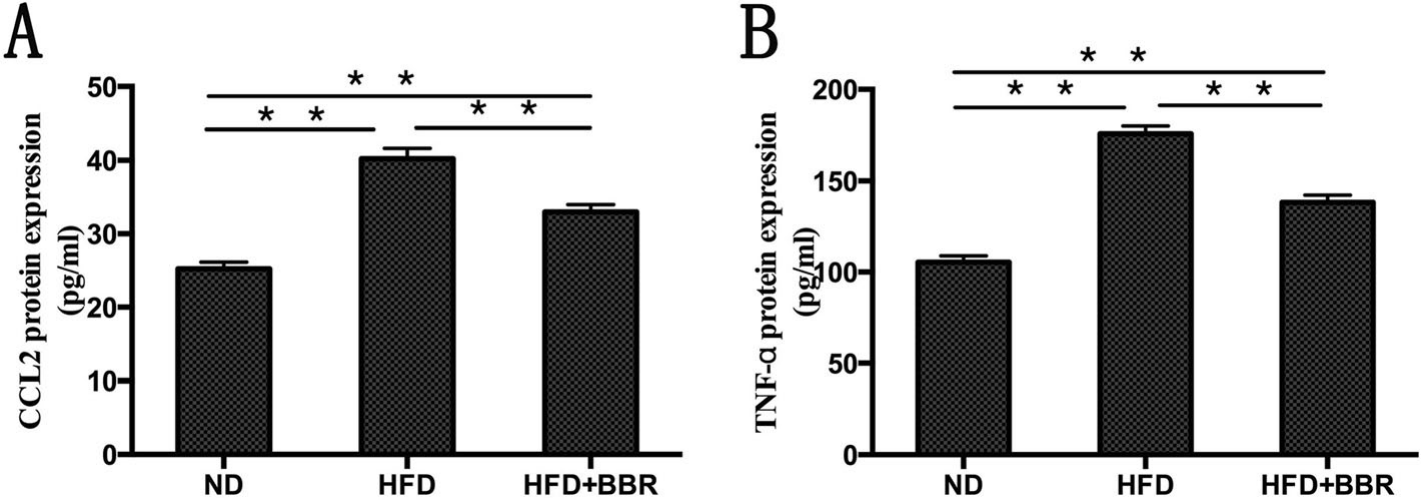
Serum proinflammatory cytokines in NAFLD rats, which were measured by ELISA. **a** serum CCL2 levels; (**b**) serum TNF-α levels. Data are presented as mean ± SD from eight rats. **P < 0.01,* *P* < 0.05.n = 8/group

To verify the therapeutic effect of BBR, we examined the effect of BBR on the serum cytokines in rats with HFD-induced NAFLD. As shown in Fig. 3, compared with those in the ND group, the serum CCL2 and TNF-α levels were significantly increased in the HFD group (*P* < 0.01). After the intervention with BBR, the serum CCL2 and TNF-α levels in the HFD + BBR group were significantly decreased compared with the HFD group (*P* < 0.05).

### Angptl2 pathway-related mRNA expression in the liver tissues of NAFLD rats

**Fig. 4:**
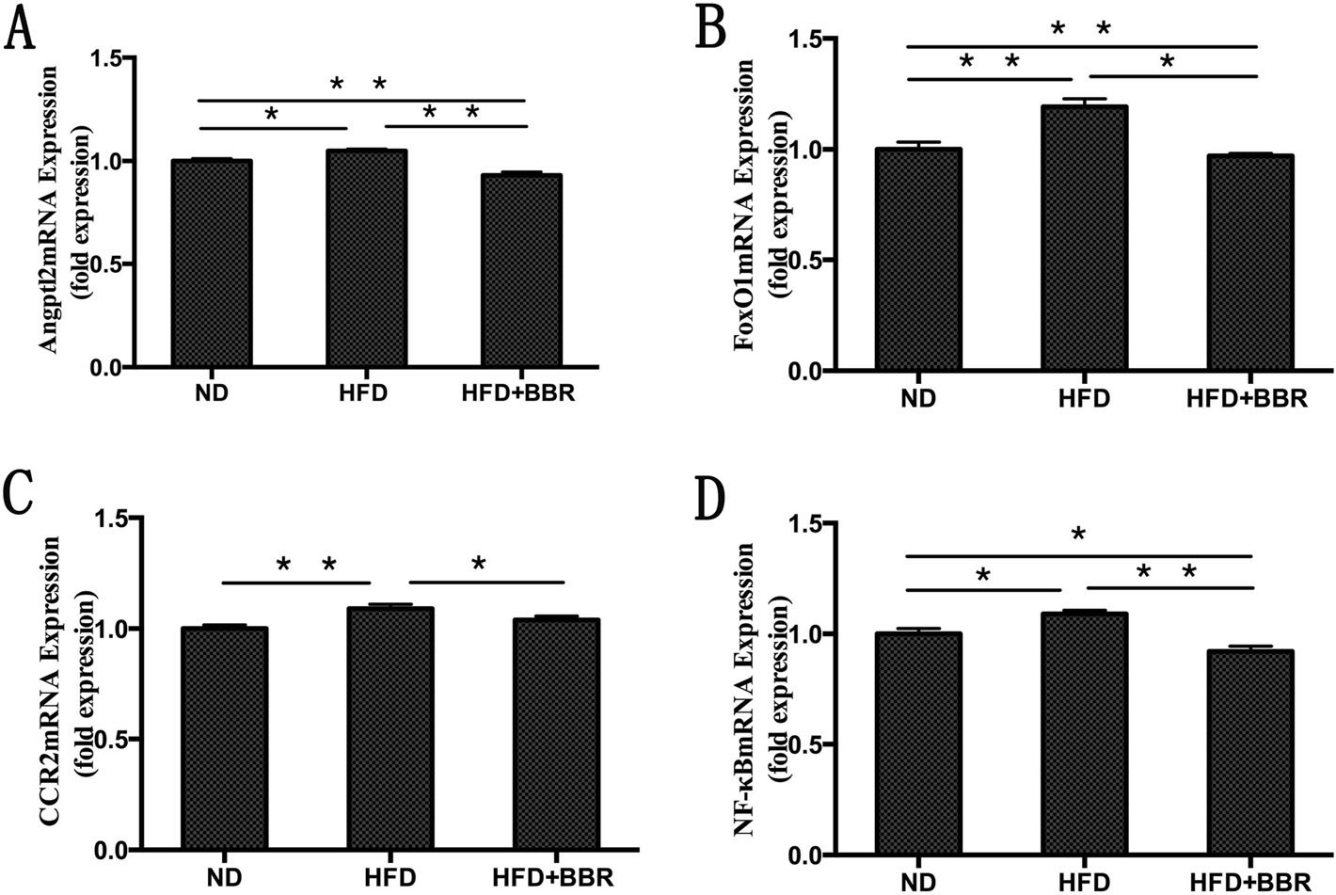
Levels of Angptl2, Foxo1, CCR2 and NF-κB-mRNA were measured by real time-PCR and normalized by GAPDH expression; **P < 0.01,* *P* < 0.05.n = 8/group

The levels of Angptl2, Foxo1, CCR2 and NF-κB mRNA in the HFD group showed different degrees of increase (Fig. 4). However, the levels of Angptl2, Foxo1, CCR2 and NF-κB mRNA in the HFD + BBR group were significantly secreased at 12 weeks compared with those in the HFD group (Fig. 4).

### Angptl2 pathway-related protein expression in the liver tissues of NAFLD rats

**Fig. 5:**
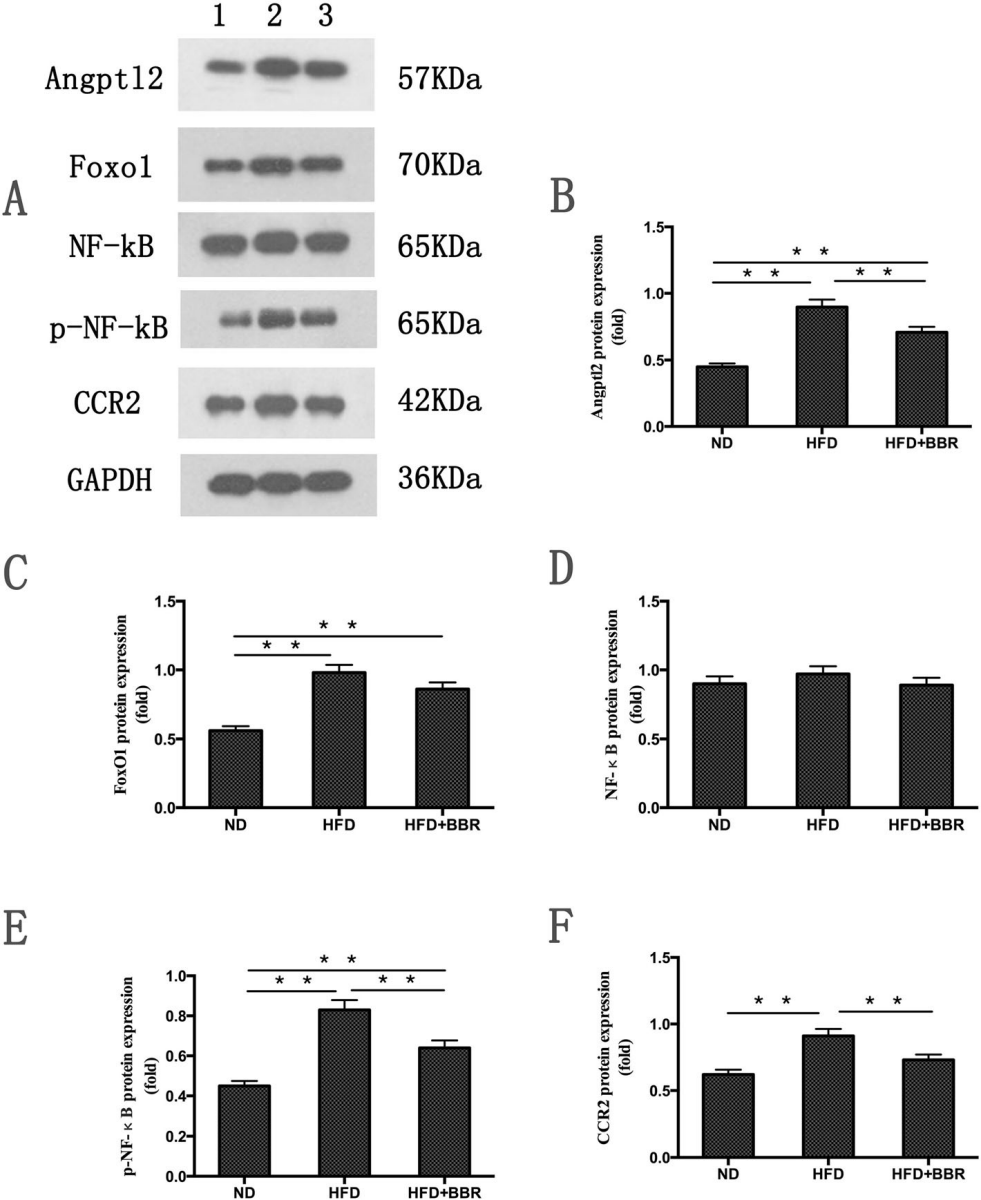
The protein levels of Angptl2, Foxo1, NF-κB, p-NF-κB and CCR2 were measured by Western blot analysis and normalized by GAPDH expression. Data are presented as mean ± SD from eight rats. **P < 0.01,* *P* < 0.05.n = 8/group

We examined the protein levels of Angptl2, Foxo1, NF-κB, p-NF-κB and CCR2 by using Western blot analysis. As shown in Fig. 5b, c, e and f, the protein levels of Angptl2, Foxo1, p-NF-κB and CCR2 were significantly increased compared with the those in ND group. An increasing trend in the liver protein levels of NF-κB was observed in the HFD group, although no statistically significant difference was observed between the HFD group and ND group (Fig. 5d). After the intervention with BBR, we found that the protein levels of Angptl2, P-NF-κB and CCR2 showed remarkable decreases compared with those in HFD group (Fig. 5b, e and f). We also observed a decrease in the liver protein levels of Foxo1 and NF-κB in the HFD + BBR group compared with those in the HFD group, but these trends were not significant (Fig. 5c and d).

## Discussion

Approximately 25% of patients with NAFLD have NASH, which is characterized by inflammation, hepatocyte ballooning, and hepatocyte injury [21]. The pathogenesis of NASH has not been clearly eluciated. The “two-hits” theory proposed by Day et al. is widely accepted by the majority scholars [22]. The first hit is “fatty degeneration”, in which lipotoxicity-induced mitochondrial dysfunction and hepatocyte steatosis make the liver more prone to inflammation under the action of the second hit. The second hit is enhancement of liver lipid peroxidation and an increase of FFAs. Although this theory does not explain all of the problems, it can be surmised that chronic inflammation plays an important role in the progression of NASH, and thus reducing the liver inflammatory response may be a potential target for treatment of NASH.

Adipocyte, macrophage-derived Angptl2 is a key mediator linking obesity and the related metabolic diseases to the inflammatory response in adipose tissues [17]. Several recent studies have shown that high-fat diet-induced obesity and the related metabolic diseases in the visceral adipose tissue of mice express a large amount of Angptl2, which significantly promotes macrophage aggregation and proinflammatory cytokine expression [23], leading to chronic inflammation and systemic insulin resistance of adipose tissue [24].AP2-Angpt12 mice (adipose tissue- specific promoter Angptl2, AP2-Angpt12) showed a marked increase in inflammatory cytokines (CCL2, and TNF-α) and inflammatory cell surface markers (CCR2) in adipose tissue [17]. In mice injected with Angptl2 by adenovirus, it was found that the accumulation of liver lipids and the expression of genes involved in fatty acid synthesis and metabolism were significantly increased, which in turn contributed to the development of fatty liver [25]. The infiltration of inflammatory cells and the chronic inflammation of adipose tissue induced by a high-fat diet in Angptl2-knockout mice were significantly reduced compared with those in wild-type mice, and glucose tolerance and insulin sensitivity were significantly increased [17]. Our study showed that the rats with high-fat diet-induce NASH were characterized by significant steatosis, inflammatory cell infiltration and necrosis. In addition, serum transaminase, lipid levels, liver lipid levels, and NAS showed different degrees of increase. Furthermore, we found that the Angptl2 expression level in the liver tissue of the HFD group was significantly higher than that in the ND group. The inflammatory cascade involved the activation of NF-κB, the numbers of CCR2+ inflammatory cells in liver tissue increased, and proinflammatory cytokines, such as CCL2, and TNF-α, in the peripheral blood were significantly elevated. Therefore, according to the above results, we speculate that the Angptl2-mediated signaling pathway is involved in the development of the NASH liver inflammatory response.

However, after BBR intervention, the pathology of liver tissue in NASH rats was significantly improved, and serum transaminases, lipid levels, liver lipids and NAS were significantly ameliorated. In addition, we found that the expression level of Angptl2 in the liver tissue of the HFD + BBR group decreased, the phosphorylation level of NF-κB decreased, the numbers of CCR2+ inflammatory cells in the liver tissue decreased, and the proinflammatory cytokines, such as CCL2 and TNF-α, in the peripheral blood significantly decreased. Previous studies by other scholars have shown that BBR ameliorate NAFLD by reducing endoplasmic reticulum stress and regulating the hepatic sirtuin 1-uncoupling protein 2 and AMPK pathways [26–28]. These findings indicated that BBR may ameliorate the liver inflammatory response in NASH through the Angptl2-mediated signaling pathway.

The Angptl2 mediated signal transduction pathway, in addition for directly activating downstream inflammatory cascades and releasing inflammatory mediators, may also be involved in regulating the following mechanisms to regulate the liver inflammatory responses of NASH: ① Foxo1 is a transcription factor in the forkhead box proteins (FOX) family that negatively regulated by insulin signaling and regulates other genes to control glucose and lipid metabolism [29], oxidative stress and apoptosis in the liver [30].Foxo1 binding to the region-specific insulin response element enhancer (nt-1457, nt-1258) of the Angptl2 promoter, upregulates the expression of Angptl2 in adipocytes through the Foxo1 pathway, promotes the release of inflammatory cytokines, such as CCL2, and participates in inflammation and systemic insulin resistance [23]. It has been found that the activity and expression of Foxo1 in liver are upregulated in NASH patients, from simple fatty liver to nonalcoholic steatohepatitis [31]. Interestingly, Foxo1 knockout significantly reduces the Angptl2 mRNA expression [23]. Our study results are consistent with the literature reports. Foxo1 expression in the HFD group was significantly higher than that in the ND group. However, after BBR intervention, the Foxo1 expression level was significantly decreased. ② TNF-α upregulates Angptl2 expression through the Foxo1 pathway, further promoting inflammatory mediators expression and insulin resistance occurence [23].Nevertheless, lentiviral-mediated siRNA interference with the Foxo1 gene can inhibit the transcriptional activity of the Angptl2 promoter, and reduce the adipocyte Angptl2 expression induced by TNF-α [32]. It can be observed that the above mechanisms can activate the inflammatory cascade mediated by the Angptl2 pathway alone or in combination, which ultimately leads to the progression of NASH liver inflammation, and BBR may ameliorate the liver inflammatory response in NASH by affecting the Angptl2 signaling pathway.

In conclusion, the findings of this study show that the change in the Angptl2 signaling pathway is one of the mechanisms of the hepatic inflammatory response induced by a high-fat diet in NASH rats, and BBR may exert anti-inflammatory effects by regulating the Angptl2 signaling pathway. However, the in vitro experiments and specific mechanisms of BBR treatment of NASH liver inflammation require further study.

## Conclusions

Hepatic inflammatory response is a key rate-limiting step in the progression of nonalcoholic simple fatty liver to NASH. Angptl2 signaling pathway activation plays an important role in the liver inflammatory response induced by a high-fat diet in NASH rats. BBR may exert an anti-inflammatory effect through regulation of the Angptl2 signaling pathway. The study results offer insight into the pharmacological mechanisms of BBR in the treatment of NAFLD.

## Methods

### Animal experiments

A total of 24 male SD rats (age 6 weeks, each weighing 180-200 g) were purchased from Shanghai Sipper-BK Laboratory Animal Co. Ltd. (production license: SCXK (Hu)2013–0016). All rats were randomly divided into three groups after 1 week of adaptive feeding as follows: (i) control group (ND group,8 rats), which was normally fed with feedstuff (21.5% protein, 12.3% fat, and 66.2% carbohydrate) produced by Jiangsu Synergic Bioengineering Co., Ltd.; (ii) high-fat diet group (HFD group, 8 rats) was given high-fat diet (comprising 21.6% protein, 36.1% fat, 42.3% carbohydrate; 82.75% basal diet + 10% lard, 2% cholesterol, 0.25% bile salt, and 5% egg yolk powder; feedstuff was produced by Jiangsu Synergic Bioengineering Co., Ltd); (iii) HFD + BBR group (8 rats) was fed with high-fat diet for 4 weeks, and intragastric administration of BBR at 300 mg·kg-1·d-1 was performed for 8 weeks starting from week 5 [Berberine (HPLC grade, ≥98%) were purchased from Chengdu Herbpurify Co., Ltd. Sichuan, China]. The normal and the model groups were administered with an equal volume of 0.9% sodium chloride, exposed to continuous light and 12:12 h light/dark cycle, and provided with food and water ad libitum. All rats were sacrificed at the end of week 12, and were fasted on water for 12 h the night before treatment. Under anesthesia, blood samples were extracted intraocularly, and liver specimens were collected. The right liver lobe was extracted and fixed in 10% neutral formaldehyde for pathological observation, and the other liver tissues were stored at − 80 °C for later use. All experimental animals were anesthetized by intraperitoneal injection with sodium pentobarbital (60 mg/kg). After deep anesthesia, cervical dislocation was performed and euthanasia was performed. Animal studies were approved by the Animal Use and Care Committee of Tongde Hospital of Zhejiang Province, and were conducted in accordance with the National Research Council Guide for Care and Use of Laboratory Animals.

### Serum biochemistry and cytokines of rats with NAFLD

Triglyceride (TG), and Cholesterol (CHOL) were used to measure the TG (Shanghai DESAY Diagnostic Technology Co., Ltd., and batch number 571/044/1), and CHOL (Shanghai DESAY Diagnostic Technology Co., Ltd., and batch number 130 /036/2) content in serum as well as liver tissues, according to the kit instructions. Levels of serum alanine transaminase (ALT) and glutamic-oxalacetic transaminase (AST) were measured according to the kit instructions for ALT (Shanghai DESAY Diagnostic Technology Co., Ltd., and batch number 27012/972006/1) and AST (Shanghai DESAY Diagnostic Technology Co., Ltd., and batch number 26010/962004/1) tests. Levels of serum free fatty acids (FFAs) measured according to the kit instructions for FFAs (R&D system Co., Ltd., and batch number CK-E30208R) tests. Levels of serum cytokines, including C-C chemokine ligand 2 [CCL2, also called monocyte chemo attractant protein, MCP-1,(Shanghai westang bio-tech Co., LTD, and batch number F3760)], Tumour necrosis factor-α [TNF-α (Shanghai westang bio-tech Co., LTD, and batch number MM-0180R1)], were measured by ELISA (Bio-Ter ELX800, USA).

### Histological examination

The liver tissues were fixed with 10% neutral formaldehyde, and embedded in paraffin after ethanol dehydration. Subsequently, tissues were cut into 5-μm-thick sections, and monitored for liver steatosis and inflammation using H & E (hematoxylin and eosin) staining optical microscopy. The diagnostic criteria were based on the European guidelines for the diagnosis and treatment of NAFLD [33].

### Real time-PCR

Trizol reagent was used to extract the total RNA in liver tissues of the 24 rats. PrimeScript kit (Takara, Tokyo, Japan) was used to synthesize cDNA by reverse transcription in a 20 μL system. The total RNA was placed in an automatic water bath at 37 °C for 15 min, and 85 °C for 30 s according to the instructions. RT-PCR was performed by ABI 7900 PCR instrument and SYBR Premix EX Taq (Takara, Tokyo, Japan). The 20 μL of PCR reaction system contained 2 μL cDNA sample, 0.4 μL (10 μM) specific upstream and downstream primers, and 10 μL SYBR Premix EX TaqII (TliRNaseH Plus) (2×), 0.4 μL ROX Reference Dye (50×), as well as 6.8 μL RNase-free ddH_2_O. RT-PCR primers were synthesized by Sangon Biotech (Shanghai) Co., Ltd. (Table 1.). The PCR reaction conditions involved denaturation at 95 °C for 3 min, 95 °C for 5 s, and 60 °C for 45 s, respectively. The PCR included 40 cycles of amplification, and each sample had three wells, with GAPDH as internal reference. Furthermore, 2^-ΔΔCt^ was used to calculate the relative gene expression.

**Table 1:** The real time-PCR primers for the validated genes

Gene		primers
Angptl2	forward	5′- GCA GGA GAG AAG AGG TTT CAA G − 3′
	reverse	5′- TCC AAG CCA CCA GTA AGT CAT A − 3′
Foxo1	forward	5′- CAG GCC GGA GTT TAA CCA GT − 3′
	reverse	5′- CTC GCT CTC TTC TAG CAG GC −3’
CCR2	forward	5′- ACC CTG TTT CGC TGT AGG ATT −3’
	reverse	5′- AAG TGC ATG TCA ACC ACA CAG −3’
NF-κB	forward	5′- GGC CTC ATC CAC ATG AAC TT −3’
	reverse	5′- TAA TGG CTT GCT CCA GGT CT −3’
GAPDH	forward	5′- GAC AAC TTT GGC ATC GTG GA −3’
	reverse	5′- ATG CAG GGA TGA TGT TCT GG −3’

### Western blot analysis

Total protein was exacted from liver tissue by using Lysis buffer containing phosphatase inhibitors (100 mM), protein enzyme inhibitor (1000 mM) and PMSF (100 mM). Protein concentrations were determined using a BCA protein quantitative kit (Applygen Co., Ltd., China). The proteins were electrophoreses in 10% sodium dodecyl sulfate-polyacrylamide gels and transferred onto polyvinylidene difluoride membranes. The membranes were blocked with 5% bovine serum albumin for 1 h. The memebrane were the incubated at 4 °C overnight with primary antibodies against Angptl2, Foxo1, NF-κB, p-NF-κB, CCR2 and GAPDH (information of these antibodies are shown in Table 2). The membranes were washed and incubated with horseradish peroxidase-conjugated immunoglobulin G antibody (Proteintech Group, China) at 37 °C for 1 h. Signals were detected through the chemiluminescent reaction by using a gel imaging system (FCE ProteinSimple chemiluminescent imaging system, ProteinSimple, USA). The relative expression levels of the target proteins were normalized against that of the GAPDH protein.

**Table 2:** Information of antibodies used for western blots

Antibody name	Catalog number	Vendor	Working concentration
Angptl2	12,316–1-AP	Affinity Biosciences	1:500
Foxo1	DF7110	Affinity Biosciences	1:1000
NF-κB	AF5006	Affinity Biosciences	1:1000
p-NF-κB	Ab3266	Affinity Biosciences	1:1000
CCR2	DF2711	Affinity Biosciences	1:1000
GAPDH	60,004–1-lg	Proteintech Group	1:5000

### Statistical analysis

All data were analyzed using SPSS17.0 software (SPAA Inc., USA). Results were expressed as mean ± standard deviation and one-way analysis of variance was used. Comparison among groups used LSD analysis (homogeneity of variance) or Dunnett’s T3 (heterogeneous variance). *P* < 0.05 was statistically significant.

## Data Availability

The datasets used and /or analysed during the current study available from the corresponding author on reasonable request.

## Abbreviations

NAFLD: Nonalcoholic fatty liver disease
NASH: Non-alcoholic steatohepatitis
BBR: Berberine
Angptl2: Angiopoietin-like protein 2
KCs: Kuffer’s cell
TG: Triglyceride
CHOL: Cholesterol
ALT: Alanine transaminase
AST: Glutamic-oxalacetic transaminase
FFAs: Free fatty acids
CCL2: C-C chemokine ligand 2
TNF-α: Tumour necrosis factor-α
NAS: NAFLD activity score
